# What’s in a Name?

**Published:** 1868-10-15

**Authors:** 


### *EDITORIAL.*

---

### *What's in a Name?*

"*Tell me, now, that name of thine;* for a name often pleases," said old Theocritus, agreeing excellently well with Shakspeare and the majority of mankind. Some men will sacrifice the best energies of a long and laborious life to secure a name. A recent novelist deplores, through a thick octavo, the miseries of a poor wretch who had *No Name.* Shakspeare, who, of course, recognizes the importance or unimportance of every and any thing, makes one of his characters use these words (which we think we have seen quoted before):

> "Good name in man and woman, dear, my lord,
> Is the immediate jewel of their souls:
> Who steals my purse steals trash; 'tis something, nothing,
> 'Twas mine, 'tis his, and has been slave to thousands;
> But he that filches from me my good name,
> Robs me of that which not enriches him,
> And makes me poor indeed."

This highly virtuous sentiment, it is true, was uttered by *Iago*, the man of somewhat dilapidated moral qualities, which detracts some little from its force; nevertheless, even the Devil can quote good Scripture, and in *apropos* style. At the present time it appears that the Devil and Iago are both wrong. In these days stealing a good name is one of the readiest means whereby little and unknown men (not to speak of some *scamps*) are foisted into notice:

> "So feeble souls are gulled,
> And they get money."

The wrongs that Sir James Clark, Sir Astley Cooper, Velpeau, Physick, Chapman, Mott, and others revered by the profession, have suffered, in this way, and the fortunes that have been built up at their expense — are they not written in the Æsculapian chronicles?

But we forbear recording them again, although no recording angel will drop a tear to blot out the record against our name of the warm expletives that rise (unspoken) to our lips. Just now we notice the organization of an "Eclectic" Medical College, which begins its career by stealing the name of the distinguished Edinburgh Professor, BENNETT, as its own patronymic. Bennett, who scorns such quackery with a depth and strength which knows no limit. Every one knows that the word *eclectic* is well enough in itself, and is the practice of every instructed physician in Christendom, but when the word itself is stolen from its legitimate signification, and then the name of John Henry Bennett is appropriated *vi et armis* — positively kidnapped, as sponsor — we — well, we begin to think the larceny passes from *petite* to *grand.* We mildly protest that the thing verges upon rascality.

Space forbids further comment just now, but we wish our readers to understand that when they ask for Prof. Gunn, of Rush Medical College, formerly of the University of Michigan, they may stumble on a person *probably* of the same name from the "*Bennett Eclectic Medical College.*"

So far as the Editor of this JOURNAL is concerned, he wishes it to be distinctly understood that he does *not* peddle electro-magnetic machines, or deal in Homœopathic pellets, Spiritualism or Uroscopy.

When physicians or patients come to this city to see any medical man of their choice, let them *beware of names*, or lending either money, time or health to strangers.

