# Erratum

**Published:** 1868-11-15

**Authors:** 


*Erratum.* In the haste of writing, on the first line of page 670, of THE JOURNAL, we inadvertently put down *Henry* instead of *Hughes*, as every one knows it should have been written. We beg J. Hughes Bennett's pardon.

